# Green Synthesis of Zinc Oxide Nanoparticles Using *Salvia officinalis* Leaf Extract and Their Photocatalytic and Antifungal Activities

**DOI:** 10.3390/biology10111075

**Published:** 2021-10-21

**Authors:** May Abdullah Abomuti, Ekram Y. Danish, Ahmad Firoz, Nazim Hasan, Maqsood Ahmad Malik

**Affiliations:** 1 Department of Chemistry, Faculty of Sciences, King Abdulaziz University, P.O. Box 80203, Jeddah 21589, Saudi Arabia; maabumoati@su.edu.sa (M.A.A.); eydanish@kau.edu.sa (E.Y.D.); 2 Department of Biological Sciences, Faculty of Sciences, King Abdulaziz University, P.O. Box 80203, Jeddah 21589, Saudi Arabia; ahmadfirozbin@gmail.com; 3 Department of Chemistry, Faculty of Science, Jazan University, P.O. Box. 2097, Jazan, Saudi Arabia; nazim7862000@gmail.com

**Keywords:** green synthesis, *Salvia officinalis*, phytochemicals, antifungal activity, photocatalytic activity

## Abstract

**Simple Summary:**

Herein, we examined the facile synthesis of ZnONPs using aqueous extract of *Salvia officinalis* without any additional stabilizing/capping agents. When compared to chemically synthesized nanoparticles, green chemistry-based synthesis using medicinal plants has less harmful effects. The photocatalytic degradation of methyl orange under UV light irradiation was performed with 92.47% degradation efficiency, and the reaction rate constant (k_app_) was found to be 0.02134 min^−1^. In addition, the antifungal activity of biofabricated ZnONPs was determined against *Candida albicans* isolates by standard protocols of broth microdilution and disc diffusion assay. Phytofabricated ZnONPs using *S. officinalis* were found to be more effective against drug-resistant *Candida albicans* isolates and have efficient photocatalytic properties**.**

**Abstract:**

The facile bio-fabrication of zinc oxide (ZnO) nanoparticles (NPs) is described in this study using an aqueous leaf extract of *Salvia officinalis* *L*. as an efficient stabilizing/capping agent. Biosynthesis of nanomaterials using phytochemicals present in the plants has received great attention and is gaining significant importance as a possible alternative to the conventional chemical methods. The properties of the bio-fabricated ZnONPs were examined by different techniques, such as UV-visible spectroscopy, X-ray diffraction spectroscopy (XRD), energy-dispersive X-ray spectroscopy (EDX), Fourier transform infrared spectroscopy (FTIR), transmission electron microscopy (TEM), scanning electron microscopy (SEM), and thermogravimetric/differential scanning calorimetry analysis (TGA/DTG). The photocatalytic activity of ZnONPs was investigated against methyl orange (MO) under UV light irradiation. Under optimum experimental conditions, ZnONPs exhibited 92.47% degradation of MO. Furthermore, the antifungal activity of bio-fabricated ZnONPs was determined against different clinical *Candida albicans* isolates following standard protocols of broth microdilution and disc diffusion assay. The susceptibility assay revealed that ZnONPs inhibit the growth of all the tested fungal isolates at varying levels with MIC values ranging from 7.81 to 1.95 µg/mL. Insight mechanisms of antifungal action appeared to be originated via inhibition of ergosterol biosynthesis and the disruption of membrane integrity. Thus, it was postulated that bio-fabricated ZnONPs have sustainable applications in developing novel antifungal agents with multiple drug targets. In addition, ZnONPs show efficient photocatalytic efficiency without any significant catalytic loss after the catalyst was recycled and reused multiple times.

## 1. Introduction

Nanoscience and nanotechnology have explored several new applications in biological science and biomedical research [1,2]. Active surface phenomenon and enhanced surface area of metal nanoparticles have proven efficacious in biomedical research [3]. The active surface area of nanoparticles with suitable real challenge applicability is modified using organic and biological moieties [4,5]. However, the core potential properties of metal nanoparticles are intrinsically persistent, and the surface properties can be modified according to the applications in environmental and biological science [6]. The synthesis and stabilization of metallic nanoparticles with surface modification are challenging tasks assigned to a particular application [7]. Green chemistry-based synthesis of nanoparticles using plant extracts is carried out at an ambient temperature and neutral pH, making it ideal to be more economical and environmentally friendly other than toxic chemical or physical methods. In biosynthesis or green synthesis, plant extracts are used to synthesize the metal nanoparticles using metal salts. Each nanoparticle’s surface properties are specifically based on the nature of the plant extract. Ag, Cu, Au, Pt, and Pd metal ions are widely reduced by converting various plant extracts to nanoparticles with unique surface properties. These noble metal nanoparticles conversely impose higher toxicity to the environment and have limited applications in biomedical science [8,9,10]. Metal oxide nanoparticles contain different surface-based chemical and nontoxic properties. Recently, photocatalytic semiconducting nanoparticles have been investigated for various applications, including photocatalytic and antimicrobial activities [11,12,13,14]. Zinc oxide (ZnO) bearing n-type semiconducting property has gained considerable interest, given its broad applicability in the fields of biomedical research, optics, and electronics [15,16,17,18,19,20]. ZnONPs are generally recognized as safe (GRAS) by the U.S. Food and Drug Administration (FDA, 21CFR182.8991) and are potentially applicable to treat infectious deceases [21,22,23,24,25].

Lipovsky et al. recently reported on the antifungal action of ZnONPs, claiming that when *Candida albicans* (*C. albicans*) was exposed to 100 g/mL ZnONPs, cell reduction of 97.5% was seen, and practically complete cell reduction was observed at 1000 g/mL ZnONPs exposure [26]. Stan et al. synthesized ZnO nanoparticles using three different types of plant extracts, including *Allium sativum* (garlic), *Alliumcepa* (onion), and *Petroselinumcrispum* (parsley), and studied their photocatalytic degradation properties against methylene blue under UV light irradiation [27]. Raja et al. recently reported the ecofriendly green synthesis of ZnO nanoparticles using aqueous extract of *Tabernaemontana divaricata* for photocatalytic activity against methylene blue and antibacterial activity against different bacterial strains [28]. Using aqueous leaf extract of *Dolichos Lablab L.* as a reducing and capping agent, Kahsay et al. conducted a one-pot green synthesis of hexagonal wurtzite and irregularly shaped ZnO nanostructures and studied their antimicrobial activity against *Bacillus pumilus* and *Sphingomonas paucimobilis* and reported the catalytic degradation of organic dyes, including MB, RhB, and OII [29]. Other semiconducting materials can kill Gram-negative and Gram-positive bacteria, filamentous and unicellular fungus, algae, protozoa, mammalian viruses, and bacteriophage [30]. Ahmad et al. [31] studied the effect of different forms (anatase versus rutile) of TiO_2_, their exposure durations, and dose concentrations on *C. albicans* culture.

Compared to other metal nanoparticles, ZnO nanoparticles from green synthesis are potentially effective and beneficial for clinical and environmental applications [32,33]. Numerous studies have been published using plant extract to synthesize ZnONPs such as leaf extract of medicinal plants *Cyanometra ramiflora* [34], *Hibiscus rosasinensi* [35], *Cassia fistula,* and *Melia azedarach* [36], flower extract of *Cassia auriculata* [37], and aqueous extract of fruit *Myristica fragrans* [38] were used as phytochemicals to synthesize ZnONPs. Recently, Patino-Portela et al. used garlic (*Allium sativum*) extract to synthesize ZnONPs and applied it as an antifungal agent against *Cercospora* sp. and evaluated the electrical interaction towards the cell membrane [39]. In another study, Madhumita et al. synthesized ZnONPs by *Pithecellobium dulce* peel extract and applied them against *Aspergillus flavus* and *Aspergillus niger*; however, the concentration required to reach 37.81% and 40.21% inhibition, respectively, was relativity higher [40]. Plant phytochemical extracts influence the size, shape, and surface properties of ZnO nanoparticles. Therefore, it is vital to perform a tunable synthesis of ZnONPs with the desired morphology and dimensions to explore their unveiled potential application as photocatalyst and antifungal agents. *Salvia officinalis L.* is a shrub of the Labiatae/Lamiaceae family and grows worldwide, mainly in Europe and North America [41]. Species of *S. officinalis* are native shrubs of the Middle East and the Mediterranean regions. This plant is usually used in the preparation of many foods due to its seasoning and flavoring properties. In many parts of Asia and Latin America, *S. officinalis* is used as a natural medicine to treat various disorders, such as inflammation, tremor, paralysis, hyperglycemia, rheumatism, and diarrhea [42]. The major phytochemical composition of *S. officinalis* is glycosidic derivatives (such as flavonoid glycosides, cardiac glycosides, coumarins, tannins, and saponins), steroids, terpenes/terpenoids (including sesquiterpenoids, monoterpenoids, diterpenoids, and triterpenoids), mostly found in leaves and flowers [43,44,45,46]. Maliki et al. obtained aqueous extract from leaves of *S. officinalis* and obtained a mixture of phenolics complex, flavonoids, tannins, saponin, mucilage, and alkaloids as phytochemicals components. This complex mixture is exploited as antibacterial (*E. coli*, *K. pneumoniae*, *P. aeruginosa*, and *P. aeruginosa*) and antifungal (*S. sereveseae* and *C. albicans*). For antibacterial and antifungal activities, a high quantity of the phytochemical mixture is usually required, which can be considered a limitation for inhibition of microbial growth inhibition [47]. *S. officinalis* phytochemical-based synthesized and stabilized ZnONPs can inhibit the growth of *C. albicans* even at very low concentrations and photocatalytic effects for toxic dye degradation, which is attributed to the surface effect of ZnONPs and phytochemical availability at the nano surface.

In this study, the leaves of *S. officinalis* containing various phytochemicals were used to acquire the aqueous extract and applied to prepare and stabilized ZnONPs. Phytochemicals present in the extract and their respective functional groups were used to fabricate the surface of ZnONPs. Bio-fabricated ZnONPs were successfully characterized by UV spectroscopy, XRD, FTIR, Raman spectroscopy, BET, TGA/DTG, SEM, TEM, and EDX techniques. The surface bandgap of bio-fabricated ZnONPs was calculated to be 2.96 V by Tauc plot. Furthermore, bio-fabricated ZnONPs exhibiting photocatalytic properties were applied to degrade the MO dye under UV-vis light, and the respective mechanism was explored. Antifungal activities against drug-susceptible and drug-resistant *C. albicans* isolates were assessed along with the determination of possible mechanisms of antifungal action to explore the possible applications of bio-fabricated ZnONPs.

## 2. Materials and Methods

### 2.1. Chemistry

#### 2.1.1. Materials

Zinc nitrate (Zn(NO_3_)_2_) and sodium hydroxide (NaOH) were purchased from Sigma-Aldrich Chemie GmbH (Taufkirchen, Germany) and were used without any further purification. Solvents, including methanol and acetone of 99% purity, were also purchased from Sigma-Aldrich Chemie GmbH (Taufkirchen, Germany). Double-distilled deionized water was used throughout the experiments. Leaf samples of *S. officinalis* were purchased from the local market in Jeddah, Saudi Arabia.

#### 2.1.2. Preparation of the Extract

*S. officinalis* leaves were crushed and washed with deionized water, followed by drying in an open shade. The aqueous extract of *S. officinalis* leaves was prepared by taking 5.0 g of the dried leaves and poured into a 250 mL beaker containing 200 mL of distilled water. The beaker was kept on a magnetic stirrer for heating at 60 °C for about 2 h. The mixture was held in an airtight bottle overnight and filtered by using Whatman No. 1 filter paper. The aqueous leaf extract of *S. officinalis* was used for the preparation of ZnONPs. The rest of the filtered extract was stored in a cool environment and dry place for further experimental use.

#### 2.1.3. Preparation of ZnO Nanoparticles (ZnONPs)

**Figure 1:**
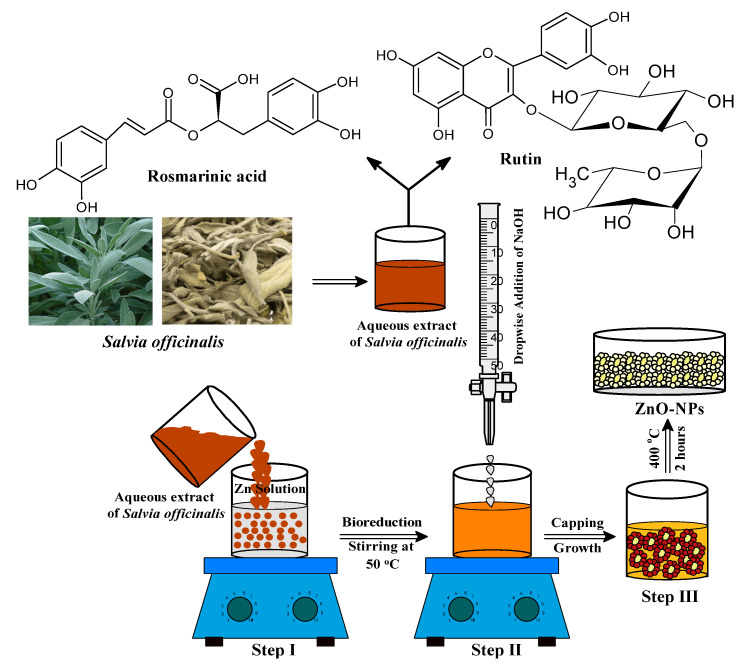
Schematic representation for the bio-fabrication of ZnONPs.

**Figure 2:**
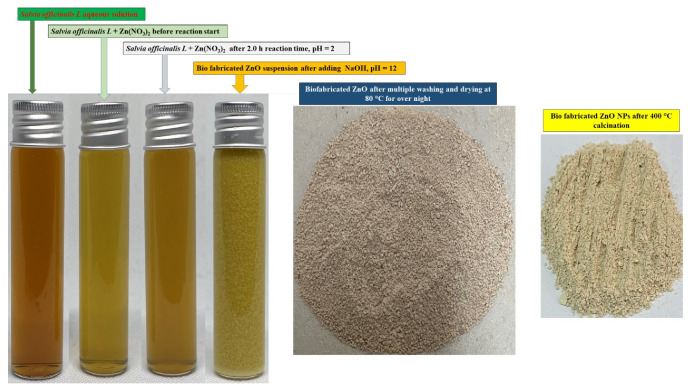
Synthesis process of ZnONPs using *Salvia officinalis* leaf extract.

Bio-fabrication of ZnONPs was initiated by taking 50 mL of an aqueous zinc nitrate solution (0.2 M) in a 250 mL beaker followed by continuous stirring at 50 °C for 10 min. The aqueous extract of *S. officinalis* (50 mL) was added to the zinc nitrate solution under constant stirring at the same temperature as shown in Figure 1 (Step I). The reaction mixture was constantly stirred for 2 h to assist the electrostatic interaction of Zn^2^^‏^^+^ with the biomolecules present in the aqueous leaf extract, and the color of the experimental mixture turned light yellow as the phytochemicals of the extract capped the Zn^2+^ ions and initiated the nucleation of ZnONPs. As the nucleation persisted, the phytochemical moieties stabilized the process, and the system reached equilibrium within the time at low pH (2.0). Solution pH from low to high enhanced the nucleation, and phytochemical moieties stabilized the ZnONPs [48,49,50,51]. In Step II, the reaction mixture (pH = 12) was maintained by adding dropwise a freshly prepared NaOH (2.0 M) solution, under continuous stirring at 50 °C. The light yellowish color precipitation occurred under constant stirring of the reaction mixture for the next 2 h at 50 °C, which indicated the formation of ZnONPs (Figure 2) [36,37,38]. The final product was collected by centrifugation of the reaction mixture performed at 10,000 rpm for 20 min. The precipitate was washed several times with ethanol and distilled water to remove any additional impurities or unreacted materials from the surface of biosynthesized material (Figure 2). In the final Step III (as shown in Figure 1), the synthesized material was kept in an oven for 24 h at 80°C to achieve complete dryness. A muffle furnace was used for annealing of the dried ZnONPs for 2 h at 400 °C. A fine light cream-colored powder of ZnONPs formed was used for further studies and surface characterization. The complete schematic representation of the bio-fabricated ZnONPs is shown in Figure 1.

#### 2.1.4. Characterization of Bio-Fabricated ZnONPs

Several techniques were used to characterize the bio-fabricated ZnONPs and analyze their thermal stability, surface, structural morphology, and optical properties. Fourier Transform Infrared spectroscopy (FTIR) (Bruker FTIR spectrophotometer) analysis performed in the range of 4000–400 cm^−1^ was used to investigate the surface chemistry and the role of phytochemical functional groups in the bio-reduction and capping of the ZnONPs. The UV-Vis diffuse reflectance spectroscopy (Thermo Scientific Evolution 600 UV-Vis spectrophotometer, Waltham, MA, USA) was used to measure the absorbance of the bio-fabricated ZnONPs in the wavelength range of 200–800 nm. Comprehensive information on the surface morphology, size, and elemental composition of the bio-fabricated ZnONPs were assimilated from transmission electron microscopy (TEM) micrographs obtained on HRTEM FEITecnai G2 30 S-Twin 300 KV system equipped with an X-ray energy dispersive spectrometer (EDX). The TEM and EDX samples were prepared by making ZnONPs suspension in ethanol, drop cast on the copper (Cu) grids, and air-dried at normal temperature. The crystalline structure, size, and purity of the as-synthesized ZnONPs were characterized through an X-ray diffraction pattern using a Powder X-ray Diffractometer (Bruker, Germany) at an operating voltage of 40 kV and a current of 30 mA supplied with Cu Kα radiation (λ = 1.5405 Å) in the 2θ range of 10–80°. The Debye–Scherrer equation was used for the size determination of the synthesized ZnONPs. Phase transition, thermal stability, and decomposition analysis were carried out by thermogravimetric analyzer (TGA-50H Shimadzu) from 30 to 850 °C at the heating rate of 10 °C/min. A spectrofluorophotometer (RF-5301PC, SHIMADZU) was used to record the photoluminescence emissions and excitation spectra at room temperature. Quantachrome surface area and pore size analyses (Nova 3200e) were used to measure the specific surface area, pore size, and pore volume distribution of the bio-fabricated ZnONPs by the Brunauer–Emmett–Teller (BET) method and the Barrett Joyner Halenda (BJH) method, respectively. Raman spectra of the bio-fabricated ZnONPs were recorded on a Bruker Raman spectrometer at room temperature using the 532 nm excitation wavelength with laser power of 50 mW to explain the molecular nature of the ZnO nanostructures.

#### 2.1.5. Photocatalytic Degradation of Methyl Orange (MO)

Dyes are the primary chemical classes used for coloring in textile industries and paper painting. These dyes cannot be easily degraded and generate aquatic problems around the world. Methyl orange (C_14_H_14_N_3_NaO_3_S) (MO) is an azo-based cationic dye, and its major side effect persists as a water pollutant with high acute oral toxicity. Bio-fabricated ZnONPs were applied to degrade the MO dye in the presence of UV light effectively. Photochemical activities of bio-fabricated ZnONPs were checked by homemade glass vessels to perform the MO dye photocatalytic reaction. Wide wavelength UV radiation was generated by 500 W mercury lamp as a light source and used to investigate the photocatalytic activities of ZnONPs against MO dye at ambient temperature and pressure. *S. officinalis*-based ZnO nanocatalysts (40.0 mg) were added to 100 mL of MO (5.0 ppm). The solution was stirred continuously for 1 h to achieve adsorption–desorption equilibrium before UV radiation. The reaction sample after stirring was kept in front of the UV radiation at 40 cm. After exposure, the samples were centrifuged (10,000 rpm for 5 min) at different definite time intervals to separate the bio-fabricated ZnONPs, and supernatants were analyzed by UV-visible spectrophotometer (Shimadzu UV-1800, Kyoto, Japan). Absorbance intensity depletions were measured at a maximum wavelength of 462 nm in the range of 200–800 nm. MO dye degradation efficiency percentage (*D*) in the presence of ZnONPs was calculated by the following Equation (1):

(1) $$D\;\;=\frac{C_{o}\;-\;\;C\;\;\;\;\;}{C_{o}}\;\times 100=\frac{A_{o}\;-\;\;A\;\;\;\;\;}{A_{o}}\;\times 100$$

where $C_{o}$ s the initial concentration and $A_{o}$ is the initial absorbance of the MO dye, while *C* is the final concentration and A is the final depletion in absorbance of the MO dye sample with bio-fabricated ZnONPs after the application of UV irradiation.

### 2.2. Biology

#### 2.2.1. Cultures and Media

Three different isolates of *C. albicans,* including one clinical fluconazole-susceptible, one clinical fluconazole-resistant, and one laboratory strain *C. albicans* SC5314, were used in this study to assess the antifungal activity of ZnONPs. All the isolates were preserved as glycerol stocks at −80 °C and were revived on Saubroad dextrose agar (SDA) plates before the experiments. All the media and reagents were procured from Sigma-Aldrich and were of high analytical grade.

#### 2.2.2. Antifungal Activity

The antifungal activity of bio-synthesized ZnONPs against three *C. albicans* isolates was determined by broth microdilution assay as per Clinical & Laboratory Standards Institute (CLSI-2008, www.clsimena.org, accessed on 22 September 2021) guidelines. Initial concentration of 1000 µg/mL of the nanoparticles was prepared using 1% DMSO (Sigma-Aldrich Co., St. Louis, MI, USA), and ZnONPs were tested within the concentration range of 250–0.125 µg/mL. All the plates streaked with *Candida* isolates and treated with ZnONPs were incubated for 24 h at 37 °C, and following incubation, minimum inhibitory concentrations (MICs) were recorded visually. Fluconazole and 1% DMSO were used as positive and negative controls, respectively.

For the determination of minimum fungicidal concentration (MFC), all the wells showing no growth were sub-cultured on SDA plates and further incubated for 24 h at 37 °C. The lowest concentration without any growth on plates was recorded as MFC.

#### 2.2.3. Disc Diffusion Assay

To further confirm the susceptibility of the ZnONPs, a disc diffusion assay was performed. All the strains were inoculated into SD broth medium and grown overnight at 37 °C. The cells were then pelleted and washed three times with distilled water. Approximately 10^5^ cells/mL were inoculated in molten agar medium at 40 °C and poured into 100 mm diameter Petri plates. Filter discs were kept on solid agar and the ZnONPs at different concentrations were spotted on the discs. Fluconazole (100 µg/mL) was also applied on the discs to serve as the positive control. The diameters of the zones of inhibition were recorded in millimeters (mm) after 48 h and were compared with that of the controls. Experiments were performed twice in replicates on separate days. The values are shown in terms of the mean ± standard error of the mean (SEM) of all three categories.

#### 2.2.4. Sterol Quantitation Method

The effect of different concentrations of ZnONPs on ergosterol biosynthesis in *C. albicans* cells was determined spectrophotometrically, using a method described previously [52]. Cells were treated with MIC, 1/2 MIC, and 1/4 MIC values for 18 h with shaking at 37 °C. After incubation, sterols were extracted using alcoholic potassium hydroxide solution and n-heptane. For analysis, samples were diluted using absolute ethanol and scanned spectrophotometrically between 240 and 300 nm using the UV-1800 SHIMADZU spectrophotometer (Shimadzu Corporation, Kyoto, Japan). Untreated cells and cells treated with 8 μg/mL of fluconazole were used as negative and positive controls, respectively. The ergosterol content was calculated as a percentage of the wet weight of the cells by the following equation:

$$\%ergosterol+\%24\left(28\right)DHE=\frac{\left(\frac{A285.1}{290}\right)\times F}{Pellet\;weight}$$

$$\%24\left(28\right)DHE=\frac{\left(\frac{A230}{518}\right)\times F}{Pellet\;weight}$$

$$\%ergosterol=\left[\%ergosterol+\%24\left(28\right)DHE\right]-24\left(28\right)DHE$$

where *F* is the factor for dilution in ethanol, and 290 and 518 are the *E*-values (in percentages per centimeter) determined for crystalline ergosterol and 24(28) *DHE*, respectively.

#### 2.2.5. Effect of ZnONPs on Cellular Morphology Using Scanning Electron Microscopy

To determine the mechanism of antifungal action of ZnONPs and their effect on cell morphology of *Candida* isolates, scanning electron microscopy (SEM) imaging was performed. The disruptive effect of ZnONPs on yeast cell morphology exposed to MIC and MFC was visualized using SEM analyzer (Zeiss EV040). Briefly, *C. albicans* SC5314 cells (1 × 10^6^ CFU/mL) were exposed to ZnONPs at MIC and MFC for 4 h at 37 °C. After exposure, both untreated and treated cells were fixed with 2 % glutaraldehyde prepared in 0.1 M phosphate buffer for 1 h at 20 °C. Following fixing, cells were washed and fixed again using 1 % osmium tetroxide for 1 h at 4 °C. For SEM analysis, samples were dehydrated in acetone, dropped on round glass coverslips with HMDS, and dried at room temperature, then sputter coated and observed under the microscope.

## 3. Result and Discussion

The aqueous extract of *Salvia officinalis* leaves contains saponins, phenolic compounds, tannins, flavonoids, alkaloids, steroids, glucosides, and proteins [38,44]. Different phytochemicals have various chemical and physical properties, including antioxidant, lipid peroxidation, and metal ion reduction and stabilization abilities. In this study, *S. officinalis* aqueous leaf extract was exploited to the capped Zn^2+^ ions, resulting in the conversion of reaction mixture into ZnONPs via nucleation followed by the stabilization of ZnONPs and their characterization using various techniques, which were then assessed for their potential photocatalytic and antifungal potential.

### 3.1. UV-Vis Spectral Analysis of Bio-Fabricated ZnONPs

**Figure 3:**
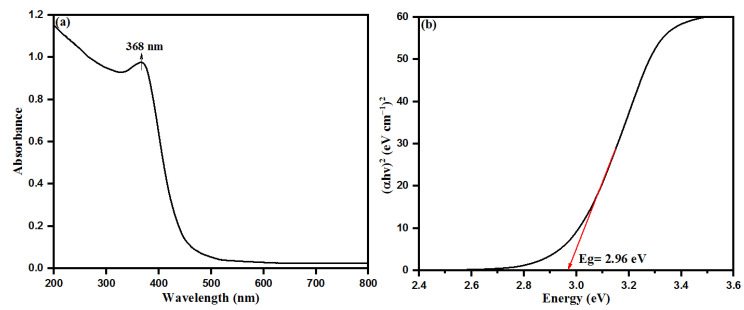
(**a**) UV−visible diffuse reflectance spectra and (**b**) Tauc plot for bandgap analysis of bio-fabricated ZnONPs.

Size and functionalization-dependent properties of nanoparticles carry a special role in changing the properties of bulky materials. Aqueous leaf extract of *S. officinalis* is a rich source of many biological moieties with high proportions of several phytochemicals, especially flavonoids and phenolics (rosmarinic acid, chlorogenic acid, ellagic acid, and luteolin 7-glucoside) [42,44,45,47]. These components possess a number of chelating and reducing properties while capping with Zn^2+^ ions and inducing the nucleation of ZnO. The flavonoid moieties initiate the capping on Zn^2+^ ions, while phenolic compounds make multi-chelating bonds and stabilize the ZnONPs after nucleation, resulting in the formation of different-sized nanoparticles. For evolution of the chemical and physical properties of semiconducting ZnONPs, UV-visible absorption spectroscopy is a widely used technique to calculate the optical properties of nanoparticles. *S. officinalis* extract-mediated ZnONPs were characterized by UV-visible spectroscopy where ZnONPs shows an absorption peak at 368 nm, as shown in Figure 3a. This absorption peak is in good agreement with earlier studies conducted on the bio-fabrication of ZnONPs using leaf extracts of *Parthenium hysterophorus* and *Azadirachta indica* [53,54]. The UV-visible spectroscopic sharp absorption peak is the confirmed evidence of stabilizing as-synthesized ZnONPs by the biological moieties present in the aqueous leaf extract of *S. officinalis*. The bandgap of bio-fabricated ZnONPs was calculated as 2.96 eV with a Tauc plot, as shown in Figure 3b.

### 3.2. XRD Analysis of Bio-Fabricated ZnONPs

**Figure 4:**
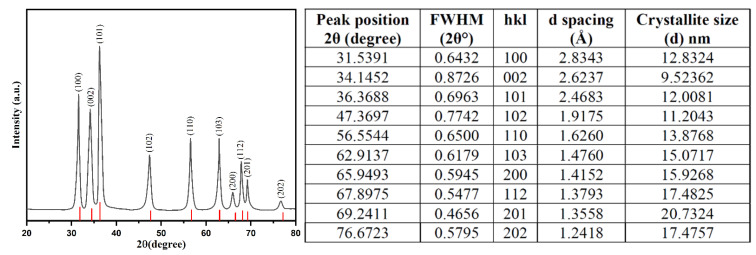
XRD pattern for the bio-fabricated ZnONPs, including X-ray diffraction data and particle size analysis.

XRD spectra analyzed the crystalline structure of bio-fabricated ZnONPs. Figure 4 shows the XRD pattern of bio-fabricated ZnONPs as confirmed by the peaks obtained with orientation planes at (100), (002), (101), (102), (110), (103), (200), (112), (201), and (002), which correspond to 2θ = 31.7°, 34.3°, 36.2°, 47.3°, 56.4°, 62.8°, 67.8°, 69.3°, 69.2°, and 76.9°, respectively. XRD data are attributed to the hexagonal wurtzite structure of the bio-fabricated ZnONPs. The observed XRD pattern revealed the polycrystalline nature of the bio-fabricated ZnONPs. Obtained data were also validated with file no. 36–1451 of the Joint Committee on Powder Diffraction Standards (JCPDS) [55]. In addition, Ali et al. also attained a similar pattern for pure ZnONPs and different amounts of C-doping in ZnONPs [56]. In our study, we observed a high intensity peak at 2θ = 76.9° for orientation peak (202) while a very low intensity peak at 2θ = 72.6° for orientation peak (004) was observed, which was attributed to the presence of C-doping in ZnONPs. The Scherrer’s formula, i.e., D = 0.9 λ/β cos θ, where “λ” is the wavelength of X-ray, β is FWHM (full width at half maximum) in radians, and θ is the diffraction angles, used for calculating the average size of ZnONPs. The average crystallite size of ZnONPs was calculated to be 11.89 nm [57].

### 3.3. Structural Morphology and Elemental Composition

**Figure 5:**
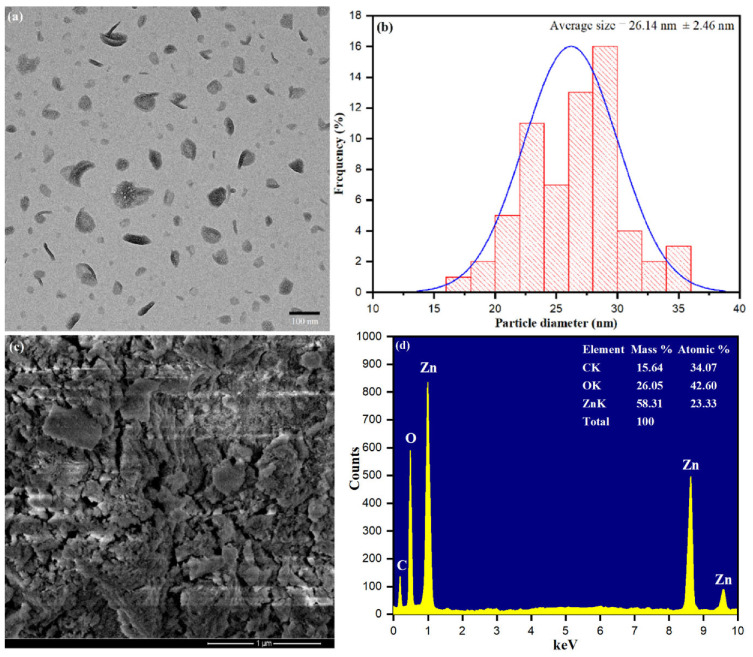
(**a**) Transmission electron microscope (TEM), (**b**) particle size distribution histogram, (**c**) scanning electron microscopy (SEM), (**d**) The EDX spectra of bio-fabricated ZnONPs.

The surface area and size of the bio-fabricated ZnONPs were characterized by TEM images. Figure 5a shows surface dispersion of ZnONPs with average size distribution. Size distributions of ZnONPs were observed and measured the average size of nanoparticles 26.14 nm, as shown in Figure 5b. The results revealed that ZnONP formation using *S. officinalis* leaf aqueous extract was stabilized by the bio-reactive components present in the aqueous medium. Figure 5c shows the SEM image, which explored some rough clumsy material surrounding the ZnONPs and a minor accumulation of clumsy material during sample preparation. Nevertheless, it was explained that the interaction of bioactive moieties influenced the accumulation of nanoparticles and stabilized their reactive surfaces for further growth in size [58]. Biologically synthesized ZnONPs were further analyzed through the EDX spectrum, as presented in Figure 5d. The EDX spectrum conformed that *S. officinalis* aqueous leaf extract-stabilized nanoparticles had high-intensity zinc metal peaks, including oxygen. In addition, carbon peaks corresponding to biological molecules such as amino acids, flavonoids, vitamins, polyphenols, and saponins were also observed.

### 3.4. TGA-DTG Analysis

**Figure 6:**
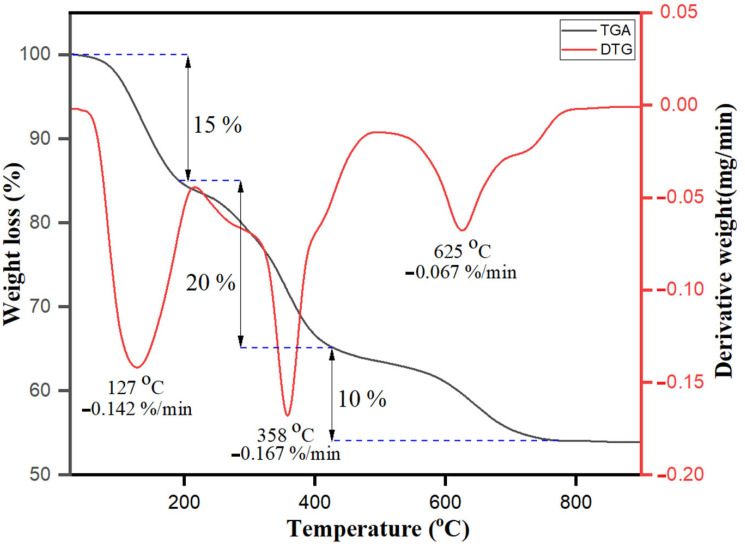
TGA−DTG analysis of bio-fabricated ZnONPs.

Thermogravimetric analysis (TGA) and derivative thermogravimetric (DTG) performance of bio-fabricated ZnONPs are shown in Figure 6. Three different significant weight loss stages were observed. The weight loss at the initial stage below 180 °C was 10%, due to water evaporation and presence of residual biological molecules on the hydrophilic organic surface moieties. *S. officinalis* extract moieties acted as a stabilizing capping agent on the surface of nanoparticles till 180 °C. The increase in temperature resulted in the enhancement in the skeleton disturbance of organic compounds and breakdown with weight loss of about 20% within the temperature range of 180–430 °C [59]. As the temperature increased, the *S. officinalis* extract-stabilized ZnONPs mimicked three respective curves, as also observed previously in dextran-coated magnetic nanoparticles [60]. Furthermore, at temperatures more than 430–780 °C, a 10% decrease in the weight loss was again observed and reached a total of 45% weight loss. Above 780 °C, no weight loss was observed. The observed data of TGA and DTG for *S. officinalis* extract emphasize that organic molecules are capable of bio-fabricating ZnONPs and have potential to act as capping agents on the surface of nanoparticles.

### 3.5. FTIR Analysis of ZnONPs

**Figure 7:**
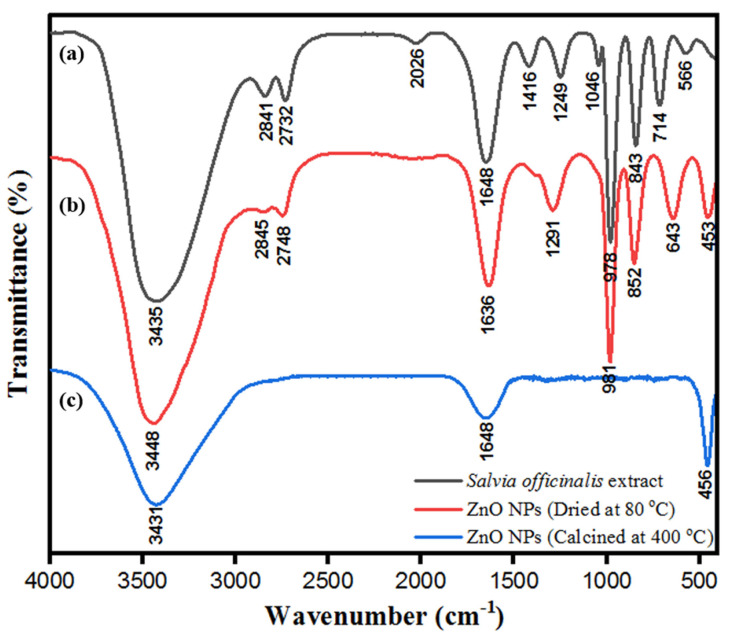
FT-IR spectra of (**a**) aqueous leaf extract of *S. officinalis*, (**b**) ZnONPs dried at 80 °C, and (**c**) ZnONPs calcinated at 400 °C.

ZnONPs were stabilized by the biological moieties present in the aqueous leaf extract of *S. officinalis*. FTIR characterization of bio-fabricated ZnONPs was performed to check the presence of expected biological moieties on the surface of ZnONPs before and after heating at 400 °C. Bio-fabricated ZnONPs were stabilized by several phenolic and flavonoid moieties present in *S. officinalis* extract, based on rosmarinic acid, chlorogenic acid, ellagic acid, and luteolin 7-glucoside. Figure 7a indicates the FTIR spectra of aqueous leaf extract of *S. officinalis*, including various characteristic peaks of polyphenols and other biomolecules at 891 cm^−1^ (-N-H, 1°, and 2° amine), 1089 cm^−1^, and 1026 cm^−1^ (*stretching -*N-H, *-*C-O, and =C-H; reflecting the presence of aliphatic amine, phenol, and carboxylic acid). The absorption band at 1290 cm^−1^ is attributed to the stretching of C-N and N-H bonds, reflecting the presence of aromatic amine phytochemicals, 1456 cm^−1^ (banding –CH_3_ and C=C), 1688 cm^−1^ (-C=O, primary amide), and broad absorption band at 3445 cm^−1^, which reflects the presence of N-H and -O-H bonds. These biological molecules might be embedded on the surface of ZnONPs, responsible for the stabilization of ZnONPs and their biological activities. After multiple washes of ZnONPs with water and ethanol, they were dried at 80 °C (dried powder shown in Figure 2) and FTIR was performed. As shown in Figure 7b, the FTIR spectrum shows ZnO absorption spectra, which reflect the exact fingerprint of biological moieties of *S. officinalis* present on the surface of the nanoparticles. These findings reinforce that bio-fabricated ZnONPs were stabilized by the biological moieties present in the aqueous leaf extract of *S. officinalis*. Later on, FTIR was performed for calcinated ZnONPs (calcinated at 400 °C). It was observed that a number of peaks disappeared, but peaks at 3431 cm^−1^ (N-H and -O-H bonds), 1648 cm^−1^ (-C=O, primary amide), and 456 cm^−1^ (Zn-O bonding) were sustained in the spectra, as shown in Figure 7c. In Figure 7b,c both spectra contain peaks at 453 cm^−1^ and 456 cm^−1^, attributed to the Zn-O bond stretching and confirming the formation of ZnONPs [38,61]. FTIR absorption-mediated fingerprint comparison of aqueous leaf extract of *S. officinalis* is shown in Table 1.

### 3.6. Photoluminescence Studies

**Figure 8:**
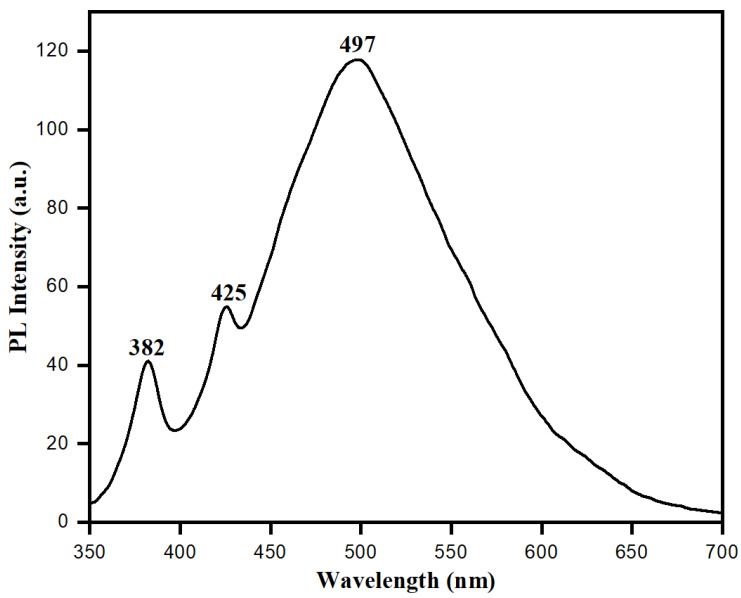
Photoluminescence spectra of bio-fabricated ZnONPs.

Distinctive electronic and optical behaviors of nanoparticles can be observed by studying their photoluminescence (PL) properties, which have valuable information about the quality and purity of material. Biologically synthesized ZnONPs showed distinct electronic and optical behaviors because of their exciton quantum confinement phenomenon and comparable size to nanoparticles or below the exciton Bohr radius. At this point, semiconducting ZnONPs may work as quantum dots which can absorb small wavelengths with an ability to emit longer wavelengths in the visible region [62]. Figure 8 shows the photoluminescence spectra of ZnONPs synthesized using aqueous leaf extract of *S. officinalis*. The photoluminescence spectra showed the presence of emission bands in the UV-visible regions. The UV emission band at 382 nm represents exciton transition. In contrast, the visible range band (green emission) at 497 nm is attributed to the recombination of photogenerated holes in ZnONPs with intrinsic defects, such as Zn interstitials or oxygen vacancies [63,64]. Photoluminescence spectra favored biological synthesis of semiconducting ZnONPs, and these nanoparticles exhibited exciton quantum confinement and stability in an aqueous medium while using *S. officinalis* extract as a reducing and stabilizing agent.

### 3.7. BET Surface Area Analysis

**Figure 9:**
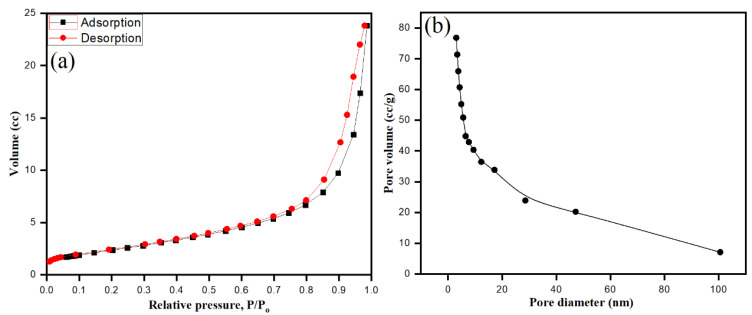
BET surface area nitrogen adsorption–desorption isotherms of ZnO (**a**), and pore diameter of bio-fabricated ZnONPs (**b**).

Bio-fabricated ZnONPs were well dispersed and stabilized by *S. officinalis* aqueous extract moieties. The surface area of ZnONPs plays a major role, adding photocatalytic and antimicrobial activities to the surface properties of the particles. Brunauer–Emmett–Teller (BET) gas sorption measurements were applied on ZnONPs, where the nitrogen adsorption–desorption process characterized the specific surface area and mesoporous features of the bio-fabricated ZnONPs at 77 K. As shown in Figure 9, ZnONPs exhibited type IV isotherm. After the application of the Barrett–Joyner–Halenda (BJH) method, mesopore sizes were determined by nitrogen desorption, while pore volume was calculated by the cumulative volume of pores [65,66]. As shown in Figure 9a,b, the bio-fabricated ZnONPs showed surface area, pore volume, and pore diameter of 53.001 m²/g, 0.240 cc/g, and 3.052 nm, respectively.

### 3.8. Raman Spectroscopy of Bio-Fabricated ZnONPs

**Figure 10:**
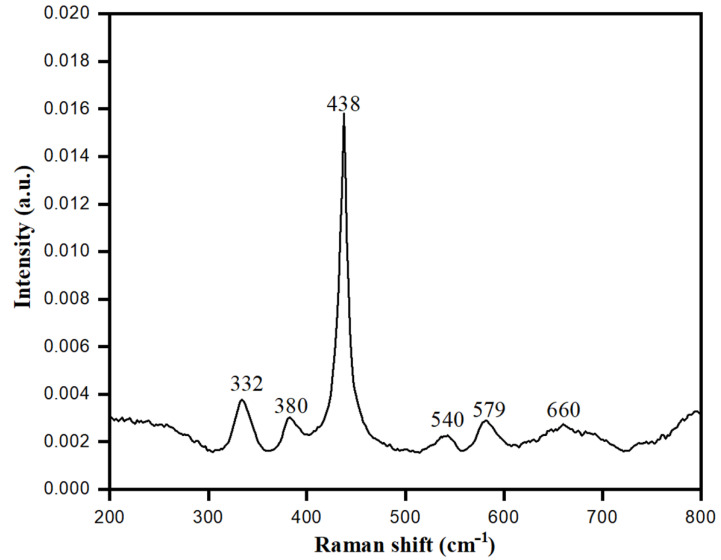
Raman spectra of bio-fabricated ZnONPs.

Crystal phases of bio-fabricated ZnONPs were analyzed by Raman spectroscopy. This technique is a non-destructive tool to acquire information about the phonon behavior of ZnONPs at different crystal phases. The shape of biologically synthesized ZnONPs was wurtzite hexagonal, belonging to space group $C_{6v}^{4}$. Based on group theory, optical phonons were A1 + 2B1 + E1 + 2E2, while according to selection rules, the modes E1+ 2A1 + 2E2 were associated with Raman, whereas A1 and E2 belonged to the optical modes of the Zn blend. These respective modes were further divided into E1 (LO) and A1 (TO) (LO, longitudinal optical; TO, transversal optical), due to perpendicular propagation towards the C axis of symmetry [67]. In the first zone of Brillouin A1 of ZnONPs crystal, E1 mode degenerated to E2 and B; however, A1 and E1 modes were generated due to Raman and IR activity while Raman activated E2 only, and B remained inactive. As shown in Figure 10, the vibrational activity of E2 (TO)-E2 (LO) mode was observed at 332 cm^−1^, whereas for A1, it was at 380 cm^−1^. The highest intensity of E2 mode was detected at 438 cm^−1^, while E1 (TO) had low intensity at 412 cm^−1^. E1 (LO) was activated and observed at 579 cm^−1^, which represented the vibration mode [68]. Raman data obtained with different modes indicated that the multi-photon resonances were present in the ZnONPs crystalline structure. From the high intensity and dominated peak of E2 mode at 437 cm^−1^ with respect to other peaks, Raman data favored the excellent crystallinity of ZnONPs synthesized using aqueous leaf extract of *S. officinalis*.

### 3.9. Photocatalytic Activities of Bio-Fabricated ZnONPs

**Figure 11:**
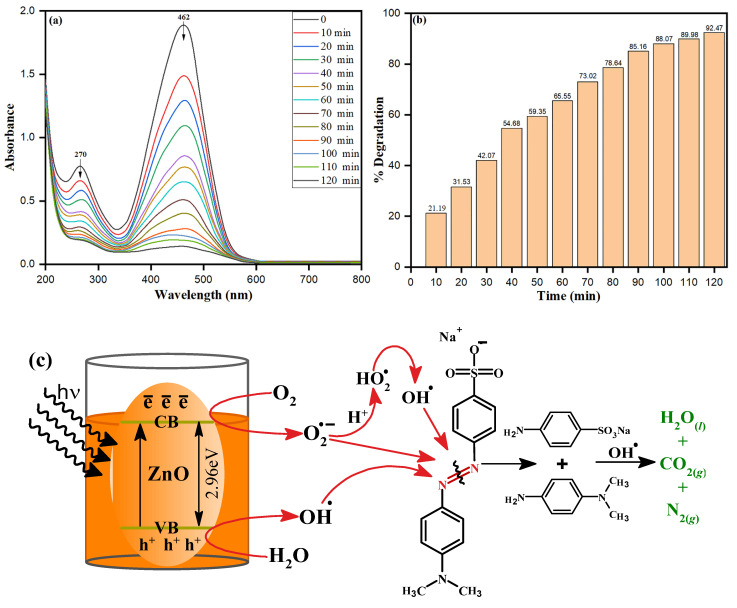
(**a**) Typical absorbance spectra of MO dye in presence of bio-fabricated ZnONPs under UV light, (**b**) MO dye degradation percentage at different time intervals, (**c**) Schematic photocatalytic mechanism of MO dye degradation under UV light.

The efficiency of as-synthesized ZnONPs on dye degradation under UV radiation was investigated using methylene orange (MO), a typical model water contaminant. Photocatalytic activity of nanoparticles is influenced by a variety of parameters, including surface area, size, radiation sources, etc. *S. officinalis*-stabilized ZnONPs were applied for photocatalytic degradation of MO dye under a wide range of UV light obtained from a 500 W mercury lamp at ambient temperature. The photocatalytic dye degradation activity of ZnONPs against MO is shown in Figure 11a. The optimum absorbance peak of MO at 462 nm was chosen to investigate the depletion in absorbance at different time zones. The high catalytic property of bio-fabricated ZnONPs was envisaged by a decrease in the 462 nm absorbance peak within 120 min. Different degradation percent values were calculated at different times. A 92.47% degradation of MO dye exposed to UV light was calculated by bio-fabricated ZnONPs after 120 min, as shown in Figure 11b. Bio-fabricated ZnONPs behaved as a semiconductor and reflected the distinct surface catalytic properties, and the mechanism can be explored based on the appropriate size of bandgap, 2.96 eV, as shown in the Tauc plot in Figure 3b. Nevertheless, the ZnONPs showed the bandgap of 3.10 to 3.37 eV with respect to different nano sizes, shapes, and other synthesis systems [69]. In our methodology, bio-fabricated ZnONPs were prepared using aqueous leaf extract of *S. officinalis* and stabilized, and they deprived the bandgap due to organic moieties present on the surface of the ZnONPs. The presence of organic doping and moieties on the surface of ZnONPs can be supported by the EDX data in Figure 5d, where carbon mass of 30.16% is shown. The bandgap also decreased due to the C doping and carbon footprint on the ZnO surface, as supported by previous research [70,71]. The small bandgap of *S. officinalis*-based bio-fabricated ZnONPs can absorb a higher visible wavelength of visible light. As photons collide with an equivalent of more energy to bio-fabricated ZnONPs, a low bandgap between the valence band and conduction band allows the generation of electron–hole pairs. These photo-generated electrons and holes move separately to the surface of nanoparticles and react with O_2_ and H_2_O to generate free radicals, as shown in the schematic representation in Figure 11c (Equations (2)–(9)). The photocatalytic degradation process of MO with bio-fabricated ZnONPs was explained based on the photogeneration of electron-hole (e^−^-h^+^) pairs between the conduction (CB) and valence (VB) bands caused by UV light excitation of ZnO [72]. The recombination of the electron–hole pair must be avoided to the greatest extent to favor a photocatalyst process, because the most critical part of this photoinduced reaction is to have reactions between the generated holes and the reductant, as well as between the active electrons and the oxidant. The photogenerated holes oxidize H_2_O and OH^−^, forming the hydroxyl radicals (OH^•^). In the meantime, the photogenerated electrons are combined with O_2_ to form superoxide radical anions (O_2_^•−^). Depending on the reaction, the O_2_^•−^ can then be protonated by H^+^ in water, generating hydroperoxyl radicals (HO_2_^•^), which can then be transformed to H_2_O_2_. For the breakdown of organic compounds, the H_2_O_2_ produced dissociates into more reactive OH^•^ radicals. Aromatic amines (phenylamine, N-methylaniline, and N,N-dimethylaniline) are then formed at early times of reaction but rapidly vanish. Finally, short-chain carboxylic acids, mainly oxalic acid and 4-aminobenzenesulfonic acid, are generated as final byproducts together with carbon dioxide, molecular nitrogen, and water as mineralization products [73].

(2) $$ZnO+h\nu \;\;\;\;\to \;\;\;\;\;ZnO\left({ē}_{\mathrm{CB}}+h_{VB}^{+}\right)$$

(3) $$H_{2}O+h_{VB}^{+}\;\to \;\;\;\;\;OH^{•}+H^{+}$$

(4) $$O_{2}+{ē}_{\mathrm{CB}}\;\;\;\;\;\to \;\;\;\;\;O_{2}^{•}\;⁻$$

(5) $$O_{2}^{•}\;⁻+H^{+}\;\;\;\;\to \;\;\;\;\;HO_{2}^{•}$$

(6) $$HO_{2}^{•}+HO_{2}^{•}\;\to \;\;\;\;\;H_{2}O_{2}+O_{2}$$

(7) $$H_{2}O_{2}+h\nu \;\;\;\to \;\;\;\;\;OH^{•}$$

(8) $$OH^{•}+MO\;\;\to \;\;\;\;Degradation\;Products$$

(9) $$O_{2}^{•}\;\;⁻+\;\mathrm{MO}\;\to Degradation\;Products$$

The free radicals react with the azo group (-N=N-) of MO dye, followed by MO degradation, leading to heavy depletion in the absorbance at 462 nm within a short time. The absorption peak of MO dye at 462 nm disappeared while the slight shift of 270 nm appeared just after 10 min of UV exposure of catalytic and MO dye, as shown in Figure 11a. Bio-fabricated ZnONPs acted intensively as catalysts under the UV irradiation to degrade orange color MO dye to colorless. Nevertheless, the ZnONPs showed 92.47% dye degradation, while in our previous research, *Trigonella foenum-graecum* seed extract-based Fe nanoparticles (2.2 eV bandgap) degraded the dye by 99.80% [74]. The comparison of the photocatalytic dye degradation of the present work with other synthesized nanomaterials is given in Table 2.

**Figure 12:**
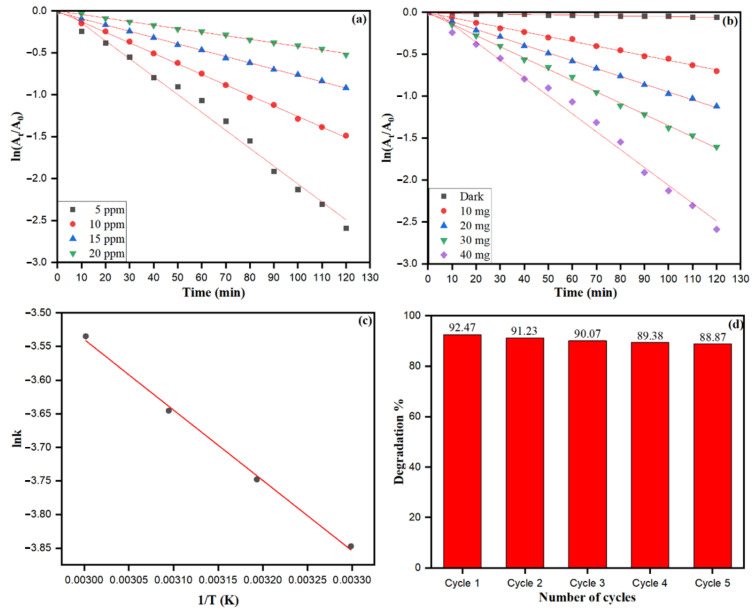
The plot of ln[AtA°] vs. irradiation time for the MO dye degradation by bio-fabricated ZnONPs (**a**) at different dye concentrations, (**b**) at different amounts of ZnONPs, (**c**) Arrhenius plot (lnk vs. 1/T curve) for MO degradation, (**d**) Effect of recycling of ZnONPs on MO dye degradation.

From the logarithmic plot of absorbance vs. time (ln A_t_/A_o_ vs. t), the degradation rate constant ‘k’ values were calculated using pseudo-first-order kinetic equation (ln A_t_/A_o_ = k), which is the simplified equation of the Langmuir–Hinshelwood kinetic model. It can be noticed that a significant amount of dye degradation (21.91% to 92.47%) at 10–120 min occurred in UV light using ZnONPs. Bio-fabricated ZnONPs attributed the highest photocatalytic activities at 40 mg treated with MO dye. The different concentrations of MO dye (5–20 ppm) were used to check the photocatalytic capability of bio-fabricated ZnONPs, as shown in Figure 12a. As the concentration of MO dye increased, the absorbance was decreased, reflecting the free radical generation on the surface of ZnONPs due to the high concentration of dye. Dye degradation rate constant (k) was calculated at different MO dye concentrations: 5 ppm (k = 0.02134 min^−1^), 10 ppm (k = 0.01256 min^−1^), 15 ppm (k = 0.00713 min^−1^), and 20 ppm (k = 0.00422 min^−1^). The photocatalytic degradation rate and rate constant of MO dye subsidized with the increase in the dye concentration. However, different amounts (mg) of bio-fabricated ZnONPs were checked for constant concentration of MO dye, as shown in Figure 12b. The MO dye degradation rate constant without ZnONPs catalyst was calculated as 0.000437 min^−1^. Nevertheless, as the amount (mg) of bio-fabricated ZnONPs increased, the rate constant (k) enhanced as 10.0 mg (k = 0.00565 min^−1^), 20.0 mg (k = 0.00933 min^−1^), 30.0 mg (k = 0.01345 min^−1^), and 40.0 mg (k = 0.02134 min^−1^). Our data showed that as the amount of bio-fabricated ZnONPs (10.0–40.0 mg) as a catalyst increased, the rate of the photoredox reaction was also increased, along with the generation of more free radicals. Figure 12c shows the effect of temperature on the MO dye adsorption and photocatalytic degradation by bio-fabricated ZnONPs; however, the rate of the photocatalytic degradation process was temperature-dependent and enhanced as the solution temperature increased. The activation energy (*E_a_*) for photodegradation of MO dye after applying the bio-fabricated ZnONPs was calculated by the Arrhenius equation at different temperatures, as shown in Equation (10).

(10) $$lnk=-\frac{E_{a}}{R}\;\left(\frac{1}{T}\right)+\;\;lnA\;\;$$

where k is the rate constant at a particular temperature, *E_a_* is the activation energy (J/mol), R is the gas constant (8.314 J·mol^−1^·K^−1^), A is the independent factor, and T is the temperature in Kelvin (K). The linear graph was obtained as its plot between lnk and $\frac{1}{T}$, and used to calculate the linear fit slope as $1.049\times 10^{3}$ K (Figure 12c). The activation energy (*E_a_*) was calculated by slope after applying the Arrhenius Equation (9), which was obtained as $8.713\times 10^{3}\;J/\mathrm{mole}$.

Bio-fabricated ZnONPs were also checked for up to five recycling stages for their applicability to degrade MO dye, as shown in Figure 12d. In every recycling stage of bio-fabricated ZnONPs, the NPs were separated from the degraded MO solution by centrifugation (10,000 rpm for 5 min). Intensive washing was performed with distilled water and the particles were dried to study the recyclability of the catalyst. We found that the application of the fifth recycle stage of ZnONPs resulted in a 3.6% reduction in MO dye degradation efficiency. The depletion of MO dye degradation after the fifth cycle may be attributed to the loss of active surface sites of ZnONPs, and biomolecules of *S. officinalis* acted as scavengers of free radicals, resulting in depletion in MO dye degradation efficiency of bio-fabricated ZnONPs.

### 3.10. Biology

#### 3.10.1. Antifungal Activity of *S. officinalis* Extract-Stabilized ZnONPs

To check the antifungal properties of bio-fabricated ZnONPs, MICs were determined against three different *C. albicans* isolates. The MIC values of ZnONPs were 1.95 and 7.81 µg/mL for fluconazole susceptible and fluconazole-resistant isolates, respectively (Table 3). On the other hand, the MIC values of fluconazole against *C. albicans* SC5314 and *C. albicans* 4175 were 0.25 and 0.125 µg/mL, respectively. In contrast, the MIC value of fluconazole against *C. albicans* 5112 was 64 µg/mL, suggesting it to be categorized as a fluconazole-resistant strain, and these findings are in accordance with the CLSI interpretive guidelines for in vitro susceptibility testing of *Candida* species [82] (CLSI 2012).

Following the MIC determination, the MFC of ZnONPs was also determined and observed to follow the same trend as that of the MICs, being most active against susceptible *C. albicans* isolates than resistant *C. albicans* isolates (Table 3). It was also observed that MFC values of ZnONPs were 2–4 times higher than MIC values. Fluconazole, being a fungistatic drug, has not been tested for MFC values.

The antifungal activities of both flavonoids and tannins have been well reported and range from 2.0 µg/mL to 30 mg/mL [83]. The high antifungal activity of the test nanoparticles in this study can be attributed to the synergistic interaction of the ZnONPs with the flavonoids and tannins attached to the surface of these nanoparticles.

#### 3.10.2. Disc Diffusion Assay

**Figure 13:**
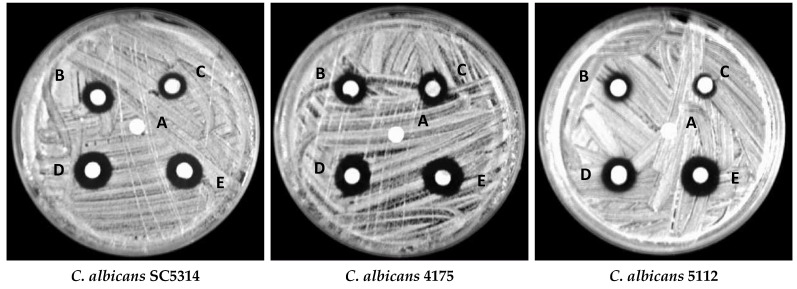
Representative images of zones of inhibition around discs impregnated with 1% DMSO (**A**), 2 µg/mL amphotericin B (**B**), ½ MIC of ZnONPs (**C**), MIC of ZnONPs (**D**), and MFC of ZnONPs (**E**) against different *Candida albicans* isolates.

To further confirm the antifungal activity of ZnONPs, a disc diffusion assay was performed. Clear zones around the discs impregnated with different concentrations of ZnONPs exhibited intense growth inhibition by ZnONPs (Figure 13). A clear inhibition zone also advocated the fungicidal activity of ZnONPs, indicating that these NPs can kill the fungi and inhibit their growth. Furthermore, from our results, it also is clear that the antifungal activity of bio-fabricated ZnONPs was in a dose-dependent fashion as the zones of inhibition increased with increasing concentrations of ZnONPs (Table 4). Comparatively, the zones of inhibition around the discs at higher concentrations were slightly bigger than the positive control amphotericin B.

The large and clear zones of inhibition by bio-fabricated ZnONPs may be attributed to the interaction of these NPs with the fungal cell wall. Biochemicals, such as flavonoids and tannins, have been reported to possess antimicrobial activity by interacting with the membrane proteins [84,85,86]. As both flavonoids and tannins were present as stabilizing agents on the surface of ZnO nanoparticles, they can directly interact with the fungal cell wall proteins, leading to the rupture of the cell wall. Flavonoids with different functional groups are major phytochemicals of plants and are well-known as antifungal agents, and used against a wide range of pathogenic fungi, including *Candida* species [87]. The hydroxyl functional group position at the phenyl ring usually determines the toxicity of flavonoids against pathogenic microorganisms. Tsuchiya et al. reported that flavonoid functional groups form a complex with extracellular proteins of the bacterial cell wall, and this complex makes the cell wall of microbial pathogens weak, resulting in sudden stop of fungal growth [88]. However, as the synthesis and processing of these NPs were subjected to high temperatures, it can be assumed that the biochemicals present in ZnONPs were degraded, and that the antifungal activity observed in this study could be due to the effect of ZnONPs.

Furthermore, ZnONPs generated reactive oxygen species (ROS), which led to suitable antifungal activities of ZnONPs towards the pathogenic fungal isolates of *Candida* species, and our results are in accordance with the study conducted by Sharmila et al. [89]. Nevertheless, the interaction of phytochemicals with fungal cell wall proteins is unknown, and this phenomenon needs to be explored in detail to further elucidate the actual antifungal mode of action of ZnONPs. The antifungal properties of bio-fabricated ZnONPs make ZnONPs a good candidate for bioactive food packaging materials. In our previous studies, we have reported antifungal activity and induction of apoptosis in *C. auris* using biogenic metallic nanoparticles [90,91].

#### 3.10.3. Ergosterol Biosynthesis Assay

**Figure 14:**
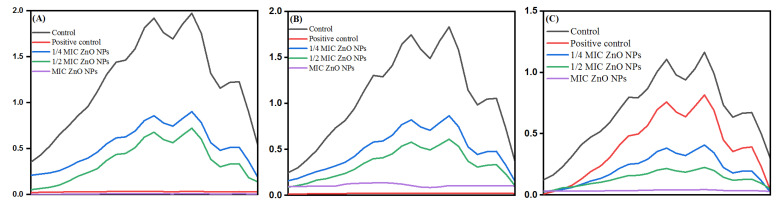
Effect of bio-fabricated ZnONPs on ergosterol levels in a laboratory strain *C. albicans* SC5314 (**A**), fluconazole-susceptible *C. albicans* 4175 (**B**), and fluconazole-resistant *C. albicans* 5112 (**C**) at MIC and sub-MIC. Untreated cells and cells treated with 8 μg/mL of fluconazole represent negative and positive controls, respectively.

After determining the MICs and MFCs of bio-fabricated ZnONPs, the effect of these nanoparticles was studied on ergosterol biosynthesis in different *C. albicans* isolates. The ergosterol biosynthesis inhibition by ZnONPs in *C. albicans* SC5314, *C. albicans* 4175, and *C. albicans* 5112 is represented in Figure 14A–C, respectively. For quantification, percentage reduction of ergosterol biosynthesis by ZnONPs in different *C. albicans* strains was calculated (Table 5). The results showed that bio-fabricated ZnONPs showed a significant decrease in ergosterol biosynthesis in a dose-dependent manner compared to untreated control cells. The percent decrease in ergosterol content at 1/4 MIC, 1/2 MIC, and MIC of ZnONPs was 59–69%, 63–74%, and 87–98%, respectively. As expected, the decrease in total ergosterol content by fluconazole (8 μg/mL) in fluconazole-susceptible *Candida* strains was above 90%, whereas in fluconazole-resistant strains, only a 17% decrease was recorded.

Furthermore, the selective efficacy of bio-fabricated ZnONPs up-thrusted the study. Although nanoparticles act by disrupting the microbial cytoplasmic membrane, specific mechanisms involved in the antimicrobial action of ZnONPs remain poorly uncharacterized. Ergosterol is a unique membrane sterol of fungi and therefore serves as an established drug target to develop antifungal drugs. Natural products, mainly comprising flavonoids, have also been shown to possess a considerable amount of antifungal activity resulting from the membrane lesion formation due to the depletion of ergosterol contents [92]. Dose-dependent inhibition of ergosterol biosynthesis in fluconazole-susceptible and fluconazole-resistant *C. albicans* isolates is in corroboration with our previous findings, where different natural and semi-synthetic compounds were reported to inhibit ergosterol synthesis in different *Candida* isolates [52,93,94]. Ergosterol plays an essential role in the physiology of *Candida* cells and is also important for the membrane heterogeneity coordination, preventing water penetration, and maintaining the integrity, rigidity, and fluidity of the plasma membrane [95]. Therefore, depletion of this sterol by the ZnONPs is directly linked to the survival of pathogens. The results of the current study provide strong evidence indicating that these bio-fabricated ZnONPs can inhibit ergosterol synthesis, thus causing fungal growth inhibition.

#### 3.10.4. Scanning Electron Microscopy

**Figure 15:**
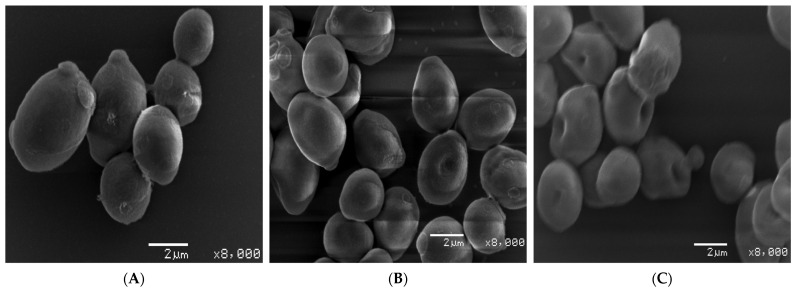
Scanning electron micrographs (SEM) of *C. albicans* SC5314: (**A**) represents untreated control cells whereas (**B**) and (**C**) represent the cells exposed to MIC and MFC of bio-fabricated ZnONPs, respectively.

After determining the effect of bio-fabricated ZnONPs on ergosterol biosynthesis, it was hypothesized that these nanoparticles may potentially disintegrate cellular envelopes in the tested *C. albicans* cells. Therefore, to study the morphological changes in cells exposed to different concentrations of ZnONPs, SEM analysis was performed. Compared to untreated control cells with smooth cell surfaces, cells exposed to MIC and MFC of ZnONPs showed wrinkling of the cell surface at varying levels (Figure 15). Alterations in cell envelopes caused by the MFC were severe compared with alterations from MIC, as is evident from the number and severity of wrinkles observed in the treatment cells.

Formation of cell membrane lesions and wrinkles, particularly after a short span of ZnONP exposure, indicates that the primary antifungal mechanism of ZnONPs involves lesion formation on the cell membrane, leading to cell death. It can also be ascertained that the observed dose-dependent fungicidal activity of these nanoparticles could be due to severe disruption of cell membrane, which can be further linked to the ergosterol depletion in the cells exposed to varying concentrations of ZnONPs. Several studies have already linked the depletion of ergosterol and membrane distribution as antifungal mechanisms. Different chemically synthesized analogs and nanoparticles have been reported to cause membrane disruption effects in different fungal pathogens [96,97]. Previous studies also reported that silver nanoparticles can disrupt the cell membranes, leading to the entry of nanoparticles into the cells, thereby reaching their drug targets [97]. In this study, we also found that ZnONPs can cause wrinkles and disrupt the cell wall and cell membrane, leading to the entry of these nanoparticles into *Candida* cells. Other researchers have also reported that the nanoparticles can cause holes and pits in *Candida* cells, thereby showing fungicidal activity against several such pathogenic fungi. Our results do not agree with these findings [98].

## 4. Conclusions

This study reports phytochemical-based biosynthesis of ZnONPs using aqueous leaf extract of *S. officinalis*. The bio-fabricated ZnONPs were characterized by using various spectroscopic and microscopic techniques for their structural characterization. The average particle size of bio-fabricated ZnONPs was 26.14 nm ± 2.46 nm with a bandgap of 2.96 eV. The bio-fabricated ZnONPs were applied successfully as a photocatalytic agent for methyl orange (MO) dye degradation under UV light irradiation. The MO dye degradation reaction was calculated as pseudo-first-order kinetics, and the reaction rate constant (k_app_) was found to be 0.02134 min^−1^. The bio-fabricated ZnONPs inhibited the growth of different clinical *C. albicans* isolates. Our study of the mechanisms of antifungal activity of ZnONPs revealed that these nanoparticles penetrate the cells by disrupting the cell membrane, thereby inhibiting ergosterol production. These findings reinforce the understanding that bio-fabricated ZnONPs have excellent photocatalytic and antifungal properties and, therefore, should be further explored in biomedical research.

## Figures and Tables

**Table 1:** Spectral positions of FTIR peaks and corresponding mode assignments of *S. officinalis* aqueous leaf extract and stabilization of ZnONPs by *S. officinalis* extract.

Peaks Assignment	*S. officinalis* in Aqueous Phase (cm^−1^)	ZnONPs Stabilized by *S. officinalis* Extract after Heating at 80 °C (cm^−1^)	ZnONPs Stabilized by *S. officinalis* Extract after Calcinated at 400 °C (cm^−1^)
*υ* (N-H) and (O-H)	3445	3438	3431
*υ_stretch_* (C-H)	2866	2874	-
*υ_stretch_* (H-C=O, C-H)	2751	2768	-
υ (C=O) amide-1°	1688	1668	1648
*υ_ben_* (-CH_3_)	1456	1416	-
*υ_stretch_* (C-N), *υben* (N-H) (Aromatic amine)	1290	1294	-
*υ_stretch_* (N-H), *υ_stretch_* (C-O) (Aliphatic amine, Phenol and Carboxylic acid)	1089	1085	-
*υ _bend_* (=C-H) (Alkene)	1026	1026	-
*υ _wag_* (N-H) (1° and 2° amine)	891	891	-
*υ_oop_* (C-H) aromatic	663	663	-
*υ_stretch_ Zn-O*	-	453	456

**Table 2:** Comparison of photocatalytic degradation efficiency of ZnO nanoparticles.

Plant Used for NPs Synthesis	Oxide NPs	Dye Used	Light Source	%Degradation	Dye Concentration (ppm)	Degradation Time (Minutes)	Ref.
*Parthenium hysterophorus*	TiO_2_ NPs	MO	Visible light source	81.5	10.0	360	[75]
*Sugar cane juice*	CeO_2_	MB	UV light	94.0	25.0	180	[76]
*Heliotropium indicum*	ZnO	MB	UV light	95.0	5.0	240	[77]
*Tephrosia purpurea*	ZnO	MB	Sun light	98.89	5.0	240	[78]
*camellia sinensis*	ZnO	MO	UV light	80.0	10.0	180	[79]
*Calotropis procera*	ZnO	MO	UV −365 nm	81.0	20.0	100	[80]
*Eucalyptus globulus*	ZnO	MO	UV light	97.0	10.0	60	[81]
*Salvia officinalis*	ZnO	MO	UV light	92.47	5.0	120	This work

**Table 3:** MIC and MFC values of bio-fabricated ZnONPs against *Candida albicans* isolates.

*C. albicans* Isolates	Fluconazole Susceptibility	ZnONPs (µg/mL)		Fluconazole
		MIC	MFC	
*C. albicans* SC5314	Susceptible	1.95	7.81	0.25
*C. albicans* 4175	Susceptible	1.95	3.91	0.125
*C. albicans* 5112	Resistant	7.81	31.25	64.00

**Table 4:** Zones of inhibition (mm) induced by bio-synthesized ZnONPs against different *C. albicans* isolates.

Concentration of ZnONPs	*C. albicans* SC5314	*C. albicans* 4175	*C. albicans* 5112
Positive control	10 ± 2	10 ± 2	8 ± 2
½ MIC	7 ± 2	9 ± 1	6 ± 1
MIC	13 ± 3	14 ± 2	11 ± 2
MFC	14 ± 2	16 ± 1	14 ± 3

**Table 5:** Effect on the ergosterol biosynthesis in *C. albicans* SC5314, fluconazole-susceptible *C. albicans* 4175, and fluconazole-resistant *C. albicans* 5112 strain after treatment with different concentrations of ZnONPs.

*Candida* Strains	Test Compounds	Mean Ergosterol Content *	
*C. albicans* SC5314	Negative control	0.0316	
	Positive control	0.0304 (96) **	
	ZnONPs	¼ MIC	0.01928 (61) **
		½ MIC	0.02118 (67) **
		MIC	0.03097 (98) **
*C. albicans* 4175	Negative control	0.0231	
	Positive control	0.02149 (93) **	
	ZnONPs	¼ MIC	0.01363 (59) **
		½ MIC	0.01456 (63) **
		MIC	0.02056 (89) **
*C. albicans* 5112	Negative control	0.02011	
	Positive control	0.00342 (17) **	
	ZnONPs	¼ MIC	0.01388 (69) **
		½ MIC	0.01489 (74) **
		MIC	0.01749 (87) **

* Expressed as a percentage of the wet weight of the cell ± standard error of the mean (followed in parentheses by the percent reduction in the mean cellular ergosterol content compared with the untreated control). ** Significant reduction compared with controls (*p* < 0.05) after Student’s *t-*test.

## Data Availability

All data is contained within the article.
